# Harnessing fluorescence for advanced characterization of textile microfibre emissions

**DOI:** 10.1038/s41598-025-27627-0

**Published:** 2025-12-16

**Authors:** Elisabeth Allen, Claudia E. Henninger, Jane Wood, Celina Jones, Andrew G. Mayes, Arthur Garforth, Eve Micklewright

**Affiliations:** 1 Department of Materials & Engineering, University of Manchester, Oxford Road, Manchester, UK; 2 Manchester Fashion Institute, Manchester Metropolitan University, Cavendish Street, Manchester, UK; 3 School of chemistry, University of East Anglia, Research Park, Norwich, UK

**Keywords:** Microfibres, Fibre fragments, Laundering, Pollution control, Fluorescence, Environmental impact, Characterization and analytical techniques

## Abstract

The rise in use of synthetic textiles, driven by low production costs and the fast fashion model, has significantly increased microplastic pollution in our environments causing both physical and chemical damage. To combat this, upstream solutions within the design and production stages of textiles is essential. However, advancements in eco-design parameters leading to the monitoring of reduced microfibre shedding are hindered by labour-intensive methods, high variability due to counting biases, and complications caused by high contamination encountered in textile testing laboratories. This research overcomes these issues by application of disperse fluorescent dye to polyester fabric before laundering, enabling detailed post-laundering microscopy analysis supported by semi-automated counting methods. The fluorescent dye penetrates the fibre matrix, enhancing the detection sensitivity of smaller, irregular fibre fragments by up to 280%. This advancement has significant implications for assessing the environmental impact of released pollutants and improving capture technologies. This methodology enables design and production parameters to be quickly and reliably analysed to address microfibre shedding. Routine analysis of microfibre shedding within the textile industry is essential for supporting legislation that will encourage more sustainable practises and ultimately mitigate the environmental impact of synthetic textiles.

## Introduction

Laundering textiles and clothing (hereon in referred to as textiles) is a significant source of environmental pollution, primarily through the emission of (micro) fibres that are released during washing. Research has identified that fibres released from synthetic textiles (such as polyester, polyamide, nylon) are one of the most common forms of air and water-borne microplastics^1,2^ with a 6 kg load of washing releasing between 700,000 microfibres per single wash^3^; release of microfibres into air is within similar magnitudes^4^. This issue is compounded by the textile production process, which often involves the use of chemicals to enhance fabric appearance and performance and thus contributing to microfibres as an emerging global problem, particularly due to their potential toxicological risks to ecosystems and human health^5^.

Despite global concern and interest in reducing microfibre release into our environments (such as the EU Strategy for Sustainable and Circular Textiles^6^, current initiatives have fallen short and focus needs to be placed on prevention through design and production processes^6–8^. Current research highlights the complex and highly varied relationship between design parameters and microfibre shedding^9–14^. To gain a greater understanding of these relationships, which could create eco-design and legislative frameworks for production, in-situ analysis within the production processes (like those used in apparel textile testing) is necessary.

However, the microscopic size of the pollution and the infancy of the research field has created analytical challenges (e.g., contamination, counting biases, fatigue) that act as barriers to accurately identify and quantify microfibers shed from textiles and apparel^15–18^. Common methods utilised within research studies, and now internationally recognised within standardised test methods, quantify microfibres shed by (1) collecting wastewater of washing effluent; (2) filtering effluents onto filter membrane; and (3) quantification of microfibres via gravimetric analysis^15,19–23^. However, these techniques do not provide size distribution information of shed microfibres, which could differ significantly between textile samples and is an important factor for overall impact and transport, behaviour and potential threat of the pollution^24^. For example, microfibres ranging from 50 μm and smaller are particularly concerning due their ability to disperse widely in air and water, increasing likelihood of being ingested by organisms. This can lead to pollutants accumulating in airways, lungs, and digestive tracts, causing physical effects such as inflammation, cross cell or tissue barriers^24,25^. In contrast, larger microfibers primarily pose risks related to chemical toxicity, as their ingestion can expose organisms to harmful substances like endocrine disruptors^24^.

Existing methodologies do not directly address contamination potential, relying instead on blank or control samples to estimate contamination, which is subtracted from the total weight of shed microfibres^3,26^. This approach introduces potential error in quantifying actual microfibre release. Gravimetric analysis alone estimates total fibre counts using equations that assume cylindrical shapes and uniform density^3,21^, but these assumptions can lead to over- or underestimation, skewing results. Additionally, this method cannot quantify the size of shed microfibres. Some studies employ imaging software (e.g., ImageJ, Fibre App) to analyse filter membranes^10,27^, while others measure individual fibres to calculate average dimensions^3,28^, combining this with gravimetric data to estimate total shed fibres. However, these techniques are time-consuming, prone to fatigue, and can introduce biases based on fibre size or colour, further affecting accuracy^15–18^.

To overcome time limitations, fluorescent stains (e.g., Nile Red) have been utilised^16,28–30^. Kang et al.^28^ report that in-situ fibre staining on the filter membrane after the washing procedure helped to “easily recognise microfibre generation” and reduced analysis time by up to 58%^29^. Nevertheless, the advantage of fluorescent staining is limited as stains can be distributed on the two ends of the fibres which can cause confusion and lead to mis-counting or lack of detection^16,31^. Additionally, as the filter membrane is subjected to the stain post filtering and processing, the fluorescent stain could also co-stain contamination and lead to over-estimating of fibre fragmentation from the test fabric, as well as staining the membrane background in some cases, reducing detection contrast^17^.

In this study, we present a detailed advancement to previously used methodologies by combining textile science with semi-automated counting technologies, to overcome existing challenges with misidentification of contaminants and to increase speed and accuracy of identification of the shed microfibres. This study examines how disperse dyes which enter the fibre matrix enhance the visibility of released microfibres, aiding analytical procedures, and in turn encouraging more sustainable design principles parameters to mitigate the release of microfibres.

## Results and discussion

**Fig. 1:**
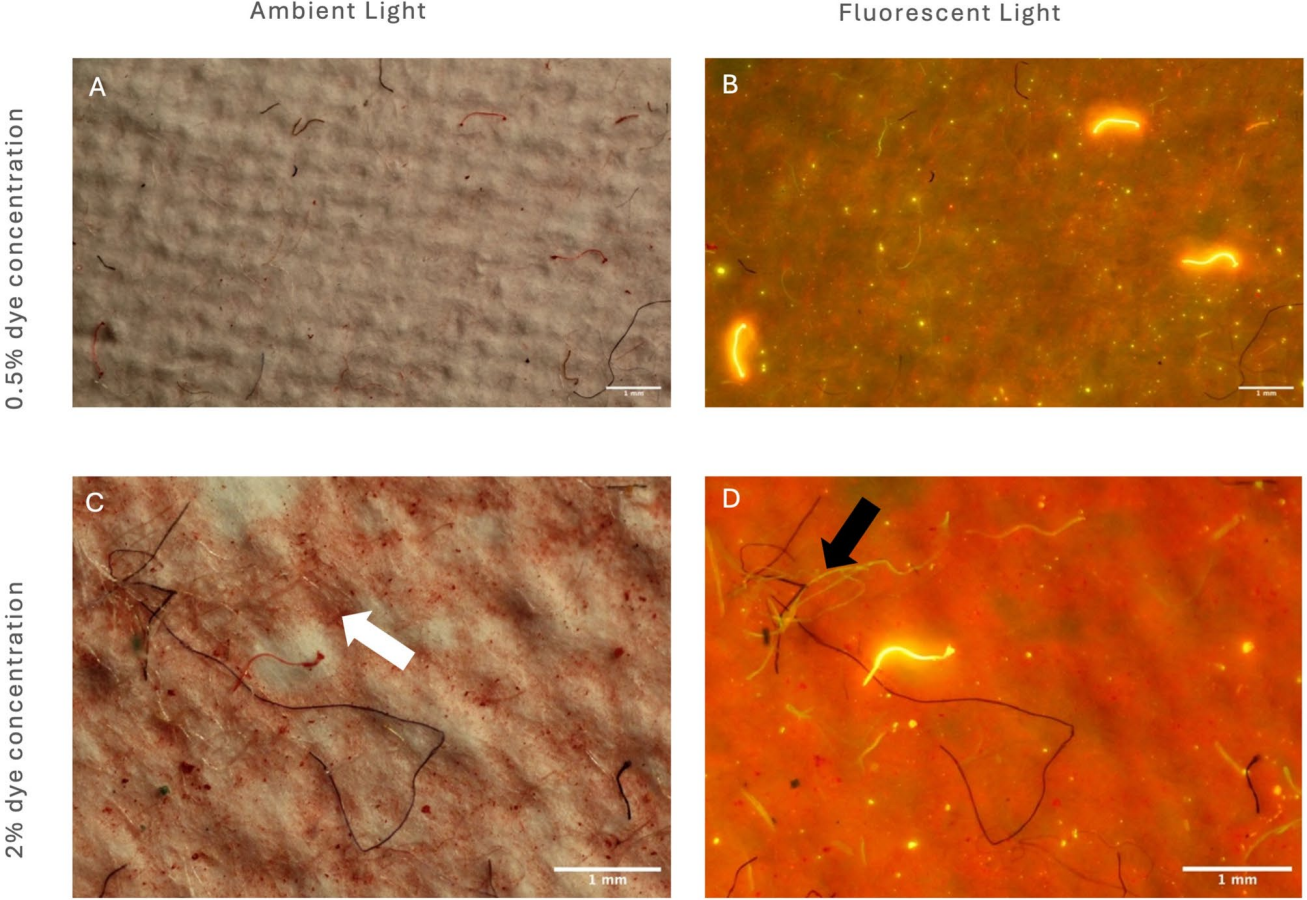
Filter membrane analysed under optical microscope at 2 x magnification for (**a**) fabrics dyed at 0.5% concentration in ambient light (**b**) microfibres shed from fabrics dyed at 0.5% concentration under fluorescent light, (**c**) microfibres shed from fabrics dyed at 2% concentration imaged in ambient light and (**d**) microfibres shed from fabrics dyed at 2% concentration imaged in fluorescent light. White arrow shows settled excess dye from fabric dyed at 2% dye. Black arrow shows procedural contamination.

**Fig. 2:**
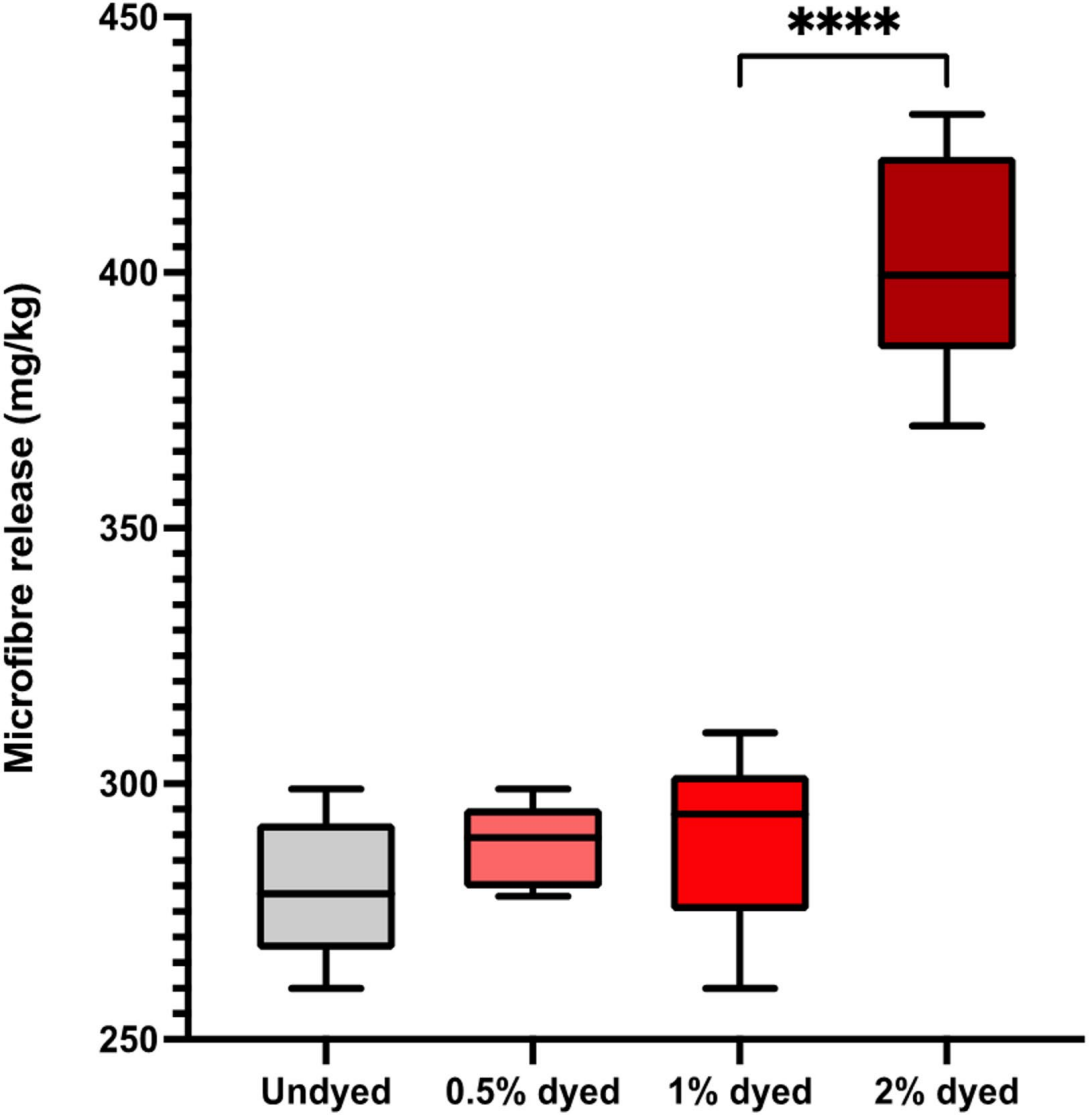
Microfibre release (milligrams of microfibres released per kilogram of fabric) for undyed fabrics, 0.5%, 1% and 2% dyed fabrics (*n* = 6).

The images show that 0.5% concentration for the dye bath is optimal due higher concentrations restricting visual analysis of shed analysis (Figs. 1C and 2).

**Fig. 3:**
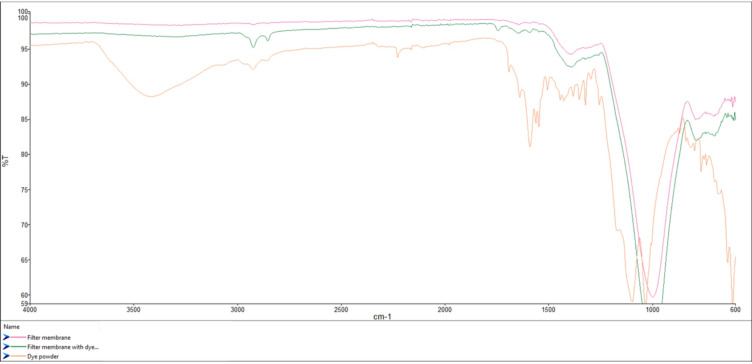
FTIR analysis of pristine un-used filter membrane, used filter membrane from 2% dye concentration (shown in Fig. 1C) and the dye powder used to dye the fabric swatches.

The undyed fabrics were subjected to the same chemical processes, and thus the results illustrate dye itself has no impact on microfibre release for fabrics dyed at 0.5% and 1.0% concentrations of the fluorescent disperse dye which showed with no significant difference (P value = 0.2067, Fig. 2). However, for the fabrics dyed at 2.0% concentration there was a significant difference from the other samples (*P* < 0.0001) which is hypothesised to be due to the excess dye on the filter membrane adding weight (Fig. 1C). To keep parameters constant between the swatches of differing dyeing concentrations to assess the microfibre release, the post-dyeing treatment of reduction clearing techniques were kept constant. Thus, it would suggest for the 2% concentration fabric swatches the reduction clearing technique was insufficient and additional or repeated reduction clearing should have been undertaken to remove all excess unfixed dye, which would remove the excess dye settling on the filter membrane and altering weights. To examine this, Fourier Transform Infrared Spectroscopy (FTIR) using Perkin Elmer Spectrum 3 with a Universal ATR Sampling Accessory (Perkin Elmer, High Wycombe, UK) examined a pristine filter membrane, the dye powder and then the filter membrane with suspected excess dye (Fig. 3).

A key advantage of this method is that when dyed under high pressure and temperature, the fluorescent disperse dye enters the fibre matrix and thus creates a full illumination of shed microfibres. This work combines the benefits of previous methods such as dyeing a bright colour to aid identification and create contrast from contamination^23,28,32,33^ as well as the use of Nile Red to improve detection rates and help combat counting biases^30,34,35^.

Previous work has dyed fabrics bright colours to aid identification of shed microfibres to contrast from common colours of contamination^23,28,32,33^. Alongside this, other work has exposed the filter membrane to fluorescent stains such as Nile Red to improve detection rates and help combat counting biases^30,34,35^. However, Nile Red has been noted for its inconsistency in staining of fibres^29,36,37^. For example, Nel et al. ^29^ suggested that particle detection rates for polyethylene terephthalate (PET, the polyester polymer) do not improve with the use of Nile Red, whilst other research highlights how Nile Red may only stain part or the ends of the fibres causing issues with miscounting of pollution released, as well as staining contamination^35,38,39^. Our work combines the benefits of both using a test fabric that is a bright colour under ambient light (Fig. 1A,C) and the benefits of being fluorescent under correct conditions (Fig. 1B, D), whilst overcoming the constraints of Nile Red inconsistencies of staining both polyester particles and fibres. Furthermore, understanding the release of polyester microfibres is necessary due to increasing evidence of uptake and movement of the smallest microplastic particles^40–42^.

An additional benefit of our methodology is that neither red nor fluorescent fibres are generally common in the lab environment, so fluorescently dyeing the fibres before the washing procedure allows clear identification of material that may have entered the water during the experiments. A conceptually similar but inverted strategy was used by Frost et al.^43^, where transparent undyed polyester fabrics were washed and thus contamination was clearly seen, as the contamination was dyed fibres which stood out. However, whilst this allowed easy identification of contamination, the undyed polyester fabrics that were laundered released transparent fibres which could be easily missed with microscopic identification^43^.

**Fig. 4:**
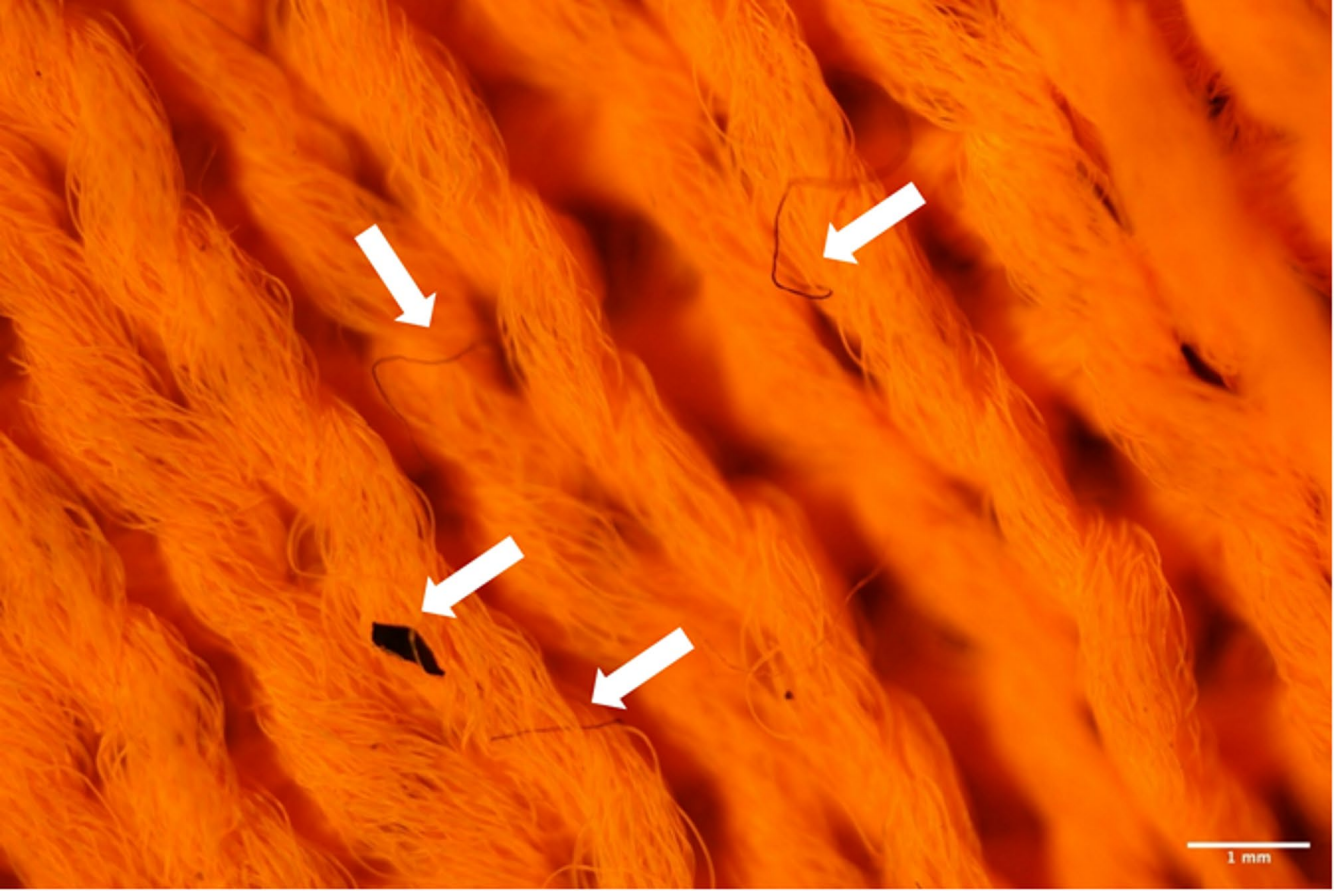
Fluorescently dyed fabric imaged under fluorescent optical microscope (1x magnification). White arrows highlight contamination that has been acquired.

The use of disperse fluorescent dyes advances on the previously used methods as it allows for easier identification of both shed fibres and allows differentiation from contamination. Figure 1B,D show that under fluorescent light the shed microfibres from the fabric swatches dyed with fluorescent disperse dye are distinguishable from procedural contamination. This is of particular importance; if monitoring of microfibre release became routine, due to the complexities of the textile industry, it could not be assumed that contamination could only be controlled within the lab. It must be accepted that levels of contamination will differ depending on processing techniques, for example, contamination embedded into the fabric structure during textile production (Fig. 4).

**Fig. 5:**
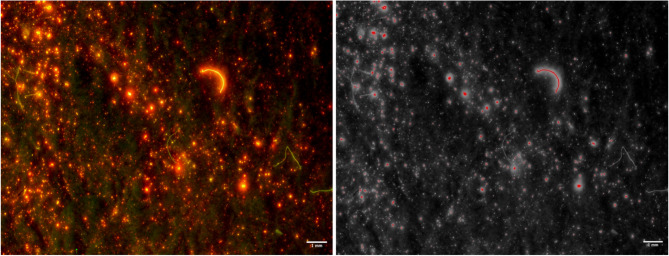
Filter membrane under fluorescent light (right), showing shed fibre fragments. Left shows the automated particle analysis tool within ImageJ using size and colour threshold restrictions.

To facilitate scalability, laboratory testing could occur in existing textile labs with high airborne contamination. Thus, methods that ensure reliable results are essential for routine monitoring, extended producer policies, and assessing the impact of textile structure and fibre characteristics on shedding (Fig. 5).

**Fig. 6:**
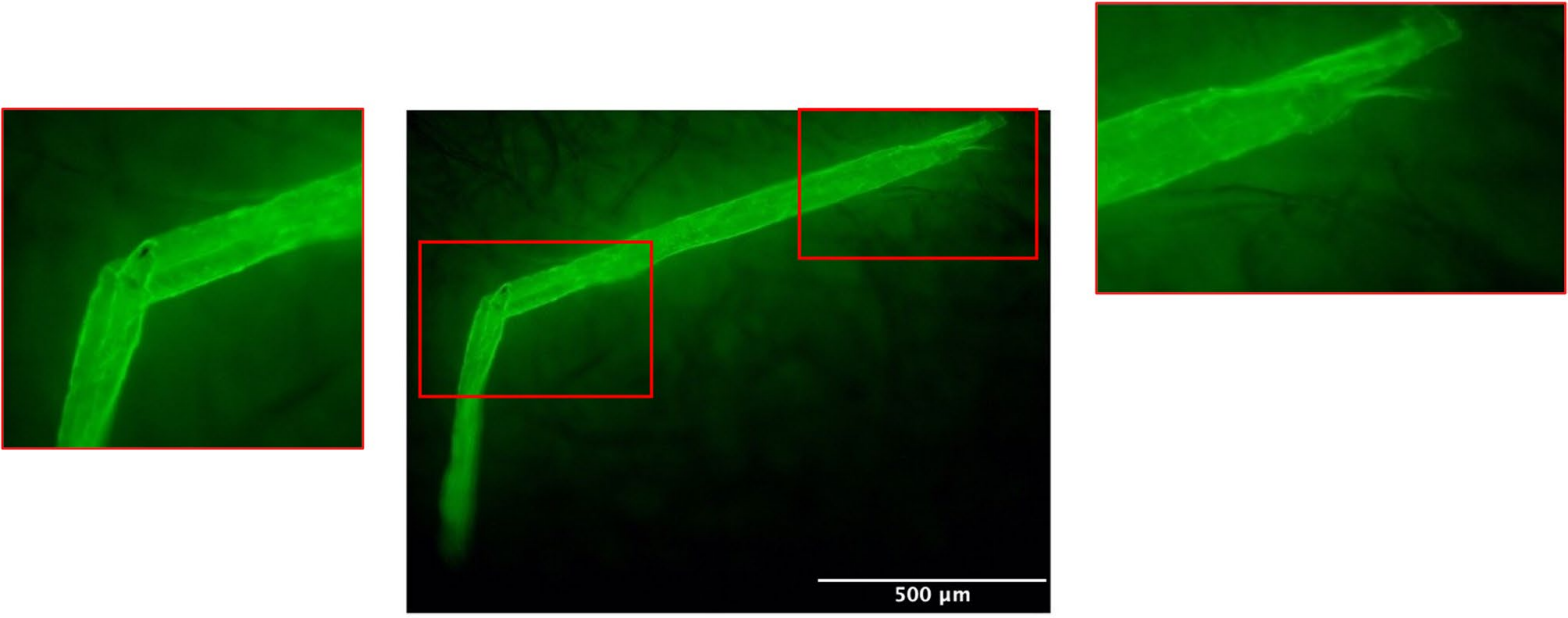
Fluorescently dyed fibre illuminating using Nikon Digital Camera DXM1200F equipment with TRITC filter cube (Ex: 540 nm).

Some fibres faintly fluoresce due to dyes and optical brighteners used in the textile industry and washing detergents, (Fig. 1D, black arrow). However, the use of software that can eliminate fibres that are not of interest allows for quicker and more precise quantification of shed microfibres (Fig. 6).

Using software such as ImageJ, and tools which allow adjustment for size and colour threshold can automate identification of particles (as known on the software) and distinguish contamination, rejecting these fibres from the count (Fig. 5). Details on exact procedure are outlined in the attached supplementary information (S1). Once selection of fibres is complete, analysis time can be further decreased with semi-automated counting using ImageJ^44,45^. This clear differentiation of shed microfibres and contamination is crucial for scaling of microfibre monitoring for work that may take place in heavily contaminated environments.

Quantification of microscopic pollutants with visual analysis can be laborious. However, with use of stains or fluorescent microscopy analysis time can be reduced by up to 58%^29^. To further decrease analysis time, a semi-automated counting technique was developed using MATLAB software (described in supplementary material, S2). Using MATLAB instead of ImageJ reduced the time required for microfibre identification, allowing the entire filter membrane to be analysed. Additionally, an increased fibre count was computed rather than manually counted. Utilising MATLAB eliminated human error and counting fatigue, consequently, more consistent results were obtained. MATLAB microfibre analysis was developed to overcome the limited “analyse particles” function on ImageJ, whereby area, perimeter and width of the bounding rectangle can be automated, but length cannot. From initial analysis of the shed microfibres under fluorescent light, it was determined that length was an important factor of this research due to the varying shape of the pollution released. For analysis using both ImageJ and MATLAB, this process is not fully automated. A threshold to identify the fluorescing fibres and fragments whilst removing background noise was established manually and could be performed in either monochrome or colour (Figure S3, S4). For future research, dependant on contamination found on the control samples, either monochrome or colour may be beneficial to ensuring contamination is not within the threshold. For this research, the main contamination identified was white and black fibres and coloured images were used to create the threshold.

Reducing analysis time is crucial to enable the quantification of all microfibres, rather than just filter sections. This is essential, as examining only small sections may fail to capture the full variability and accurate representation of microfibres shed during laundering. Additionally, microfibres may not be uniformly spread on the filter membrane^27,46,47^. The imaging equipment used allowed semi-automation of capture of the full filter image. Furthermore, this equipment can be inexpensively replicated with costings and sources outlined in Maes et al.^16^. By creating an inexpensive, easier analysis method whilst decreasing time encourages mainstream monitoring of microfibres during existing textile testing and production. Increased monitoring and better understanding of pollution released can lead to greater control through (in)voluntary policies to limit the reduction of microfibre pollution.

As described above, using the fluorescent dye and semi-automated counting software (ImageJ or MATLAB) can aid monitoring of shed microfibres. Additionally, Fig. 6 demonstrates that with the use of fluorescent disperse dye, the twist of the polyester fibres can be seen, as well as splitting of the ends of the fibres, which in certain environments may further fragment and create smaller microfibres. Future research could use this method to easily see pollution placed in different modelled environments such as air transmission models, wastewater treatment plants, marine environments, or marine organisms, establishing modes of fibre fragmentation, developing understanding of pathways, and rates of degradation^48^. Additionally, fluorescent-dyed microfibers could facilitate the identification of fibre fragments in tracking or uptake experiments involving marine organisms.

**Fig. 7:**
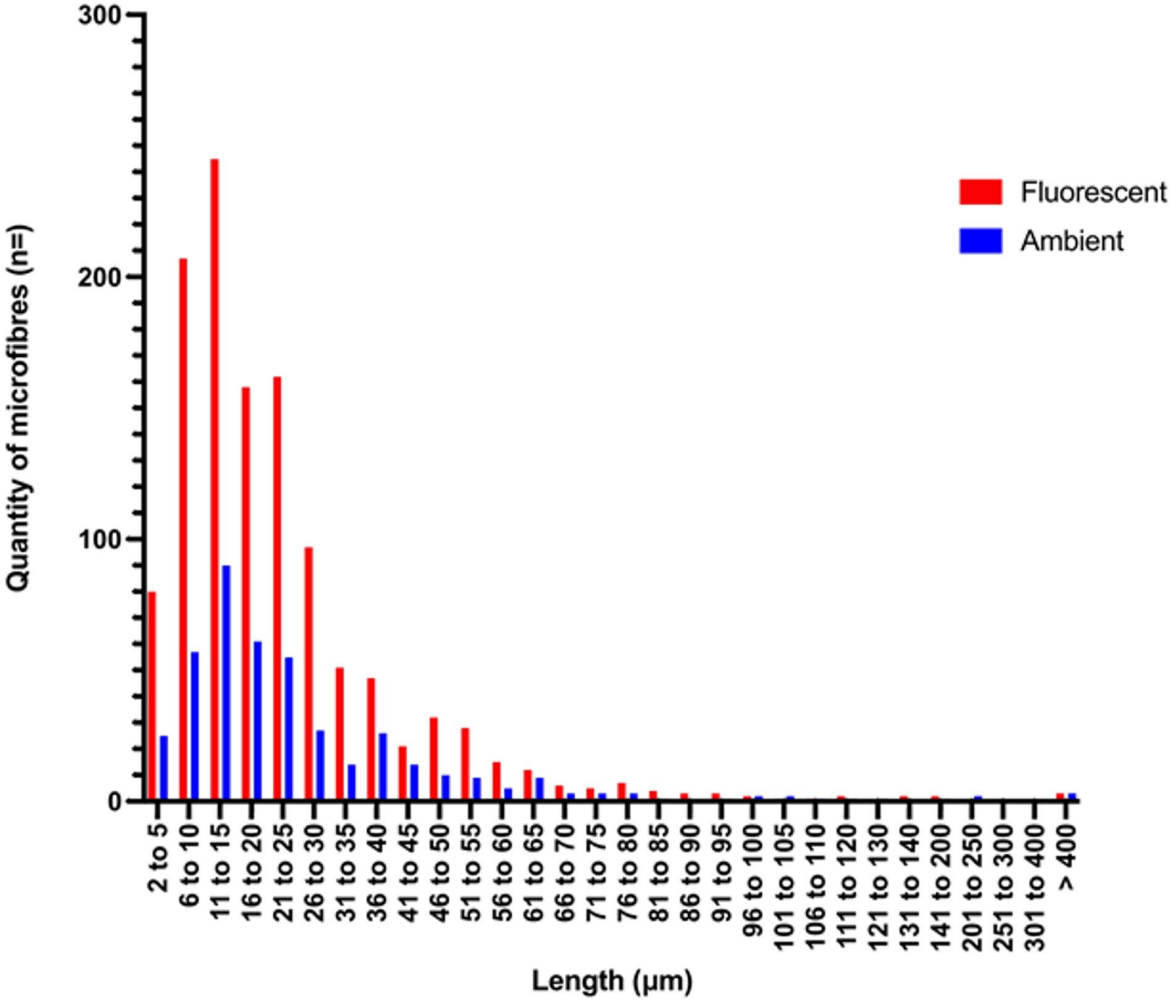
Quantity and length of microfibres (µm) identified by manual counting using ImageJ software under fluorescent light and ambient light. Lower limits of microfibre detection for digital microscope is 2 μm. Quantity of microfibres averaged over 12 images (6 fluorescent and 6 ambient).

**Fig. 8:**
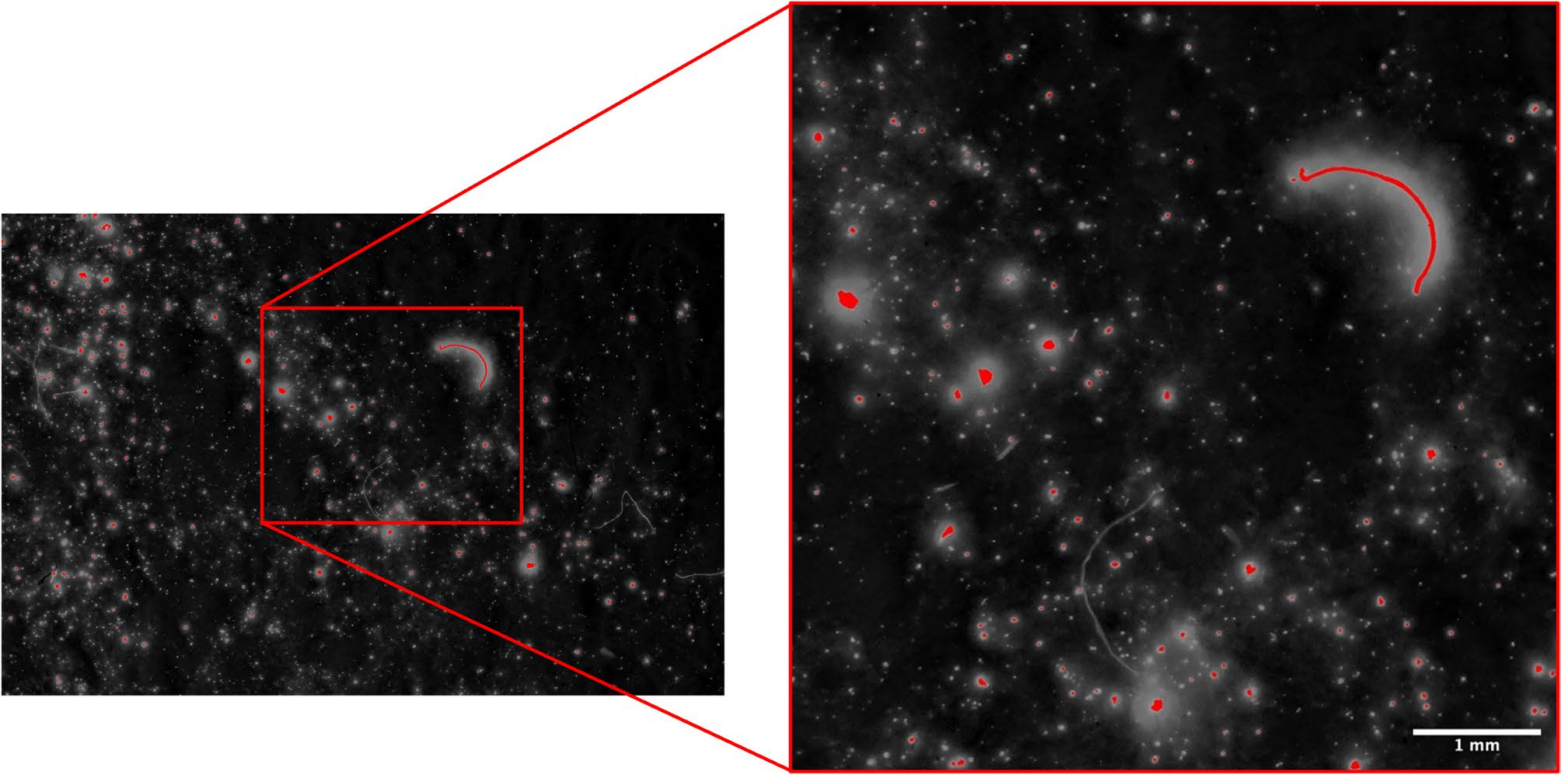
Analysis of the fluorescent microfibres released from the test fabric, highlighting the varying shapes of the pollution released.

Furthermore, the use of fluorescent disperse dyes allows more effective detection of polyester fibres, including smaller fibre fragments which may have been overlooked by other methods. Microfibre counts using ImageJ under ambient and fluorescent light (detailed in Supplementary Material, Figures S2–S9) revealed that fluorescence-based counting increased fibre identification by over 280% compared to bright dyes alone (Fig. 7). The difference in quantification methods created a statistically significant difference in amount of microfibres identified (p value < 0.004). Scaling up, using fluorescent light identified ~ 675,000 microfibres per kg of washed fabrics compared to ~ 247,000 per kg when analysed under ambient light. This aligns with findings of Kotar et al.^49^ and Mariano et al.^50^ whereby fluorescent dyes were used to identify microplastic particles and highlighted how fluorescent visual microscopy is an easy, effective and accessible tool to quantify pollution with samples of known polymer compositions. Furthermore, as illustrated by Fig. 7, a larger quantity of smaller microfibres was identified when fluorescence was utilised. Despite the differences in quantities of fibres identified, the modal group of the size of microfibres identified was the same for both fluorescent and ambient light at 11 μm to 15 μm. This is significantly smaller than the previously reported average sizes of microfibres ranging from 170 μm to 7790 μm (Table 1;^3,27^). The capability to identify smaller particles is crucial because smaller size fractions of micro-particle pollutants are particularly important for exposure and health risk assessments^49,50^.

Throughout this work, the use of disperse fluorescent dyes in the illumination of the smallest of polyester fragments released from the fabrics during laundering has been discussed. Previous research has suggested that “microplastics originating from textiles typically have a fibre shape”^51^ which can be attributed to pollutants of a shape with “high ratio of length to thickness” (pg. 455; ^52^). However, the findings of this work suggest that the released pollution may be of varying shapes, rather than cylindrical ‘fibre’ shape assumed by previous studies^3,26,51^. Historically, environmental microplastics have been categorised via shape (fibre, fragment, pellet, film, foam) which can then relate to the source of the pollution, alongside the fate and transport of the micro-pollution^53^. This work identified that most of the pollution released from textiles was of irregular shape with a low ratio of length to width, similar to ‘fragment’ categorisation rather than a cylindrical fibre shape (Fig. 8). This suggests that previous research may have missed some microfibre pollution, because the shape of the pollution would not have fit into the assumed cylindrical ‘fibre’ category. Micro pollution released from fabrics during laundering may be smaller and of varying shapes (Figs. 5, 6 and 8) due to the yarn splitting and fragmenting as a result of chemical and mechanical stress in the laundering process. Overall, this could have implications for harm caused by the pollution as decreasing size can enhance uptake of chemicals and greater ability for mobility in environments and easier ingestion into organisms^24^. The varying shapes of pollution released identified with this work further clarifies the need for continued investigation into the true scale and impact of microfibre pollution. Furthermore, current filtration techniques may be based on mitigating release of cylindrical shaped fibres and therefore further development of identification technology is necessary.

**Table 1:** Example of average length of microfibres identified from current literature. Textile parameters,, the washing test method and pore size has been highlighted as these can also have an influence on microfibre release or on the size of the microfibres collected.

Reference	Average length of microfibres	Textile	Washing test method	Isolation pore size	Analysis method
This study	11–15 μm	100% PET	Laboratory scale washing test method, 45 min at 40 °C	1.2 μm	Image processing with ImageJ
Galvão et al. ^27^	170 μm	Mixed load	Full size washing machine, commercially available washing machine	12 μm	Image processing with ImageJ
Palacios-Marín et al.^55^	550 μm	Flat 100% PET, textured 100% PET, staple 100% PET, polycotton blend (52% PET, 48% cotton) and 100% cotton – individually tested	Laboratory scale washing test method, 45 min at 40 °C	1.6 μm	Image processing with Image-Pro7
Özkan and Gündoğdu^36^	1590 μm	Recycled PET, virgin PET – individually tested	Laboratory scale washing test method, 45 min at 40 °C	0.45 μm	Image processing with ImageJ
Napper and Thompson^56^	7790 μm	Mixed load	Full size washing machine, commercially available washing machine, 45 min cycle at 30 °C	1 μm	Image processing with ImageJ

The methods in this study presented encouraging results, highlighting the use of fluorescent disperse dyes to enhance the understanding of pollution released from polyester fabrics during laundering. There are a multitude of European Environment Agency^51^ calls to improve microfibre release mitigation strategies and this work forms part of the foundation for developing the evaluation of microfibre release.

Transitioning to garments and textiles that release less pollution relies heavily on understanding of how the design and production of textiles impact microfibre shedding during the garment’s lifecycle. Furthermore, by dyeing yarn prior to fabric creation, or by dyeing the thread used to sew the fabric into garments, could allow for in-depth analysis of the fabric creation i.e., having the warp and weft or threads in different fluorescing colours would allow for subtle aspects of the shedding process to be assessed. Furthermore, using existing testing standards and analysing fabric swatches rather than full size garments is encouraged to continue gaining understanding due to being the “preferred method to understand subtle differences in fabric construction” (^8^ pg. 145) which is a key priority for future studies^9^.

In conclusion, the methods presented in this study will allow for rapid and refined analysis of microfibre shedding from textiles, which will allow a greater understanding on the complex relationship between fabric creation and microfibre shedding that can be manipulated to create garments and textiles that release less pollution. The ability to harness fluorescence Given the widespread presence of microfibres in the environment, particularly from textile laundering, enhancing methodologies to provide insights into size, toxicity potential, and extent of this pollution, is crucial^49,57,58^. Without this information, effective policies and (in)voluntary techniques to reduce microfibre pollution cannot progress.

## Methods

### Sample Preparation

Polyester fibre was chosen for this study because it is the most common fibre used in the textile and clothing market^59^. Fabrics were created specifically for this study using raw white polyester yarn, specification − 2 ply yarn, 167 dtex, 48 nm filament length, 100 turns per meter spun Z-twist (JH Ashworth, Hyde, UK). The fabric was knitted in a single jersey construction created using a 10-gauge Dubied knitting machine with a feeding tension of 10, sited at the University of Manchester. Table 2 provides more details on the fabric specifications. The fabric was heat set at 180 °C for 50 s (Beta Major pneumatic swing heat press Adkins, UK), then laser cut to cut, seal and finish the edges of the fabric (FB1500 series, CadCam Technology, UK) into 9 cm-by-9 cm swatches.

### Dye procedure

**Fig. 9:**
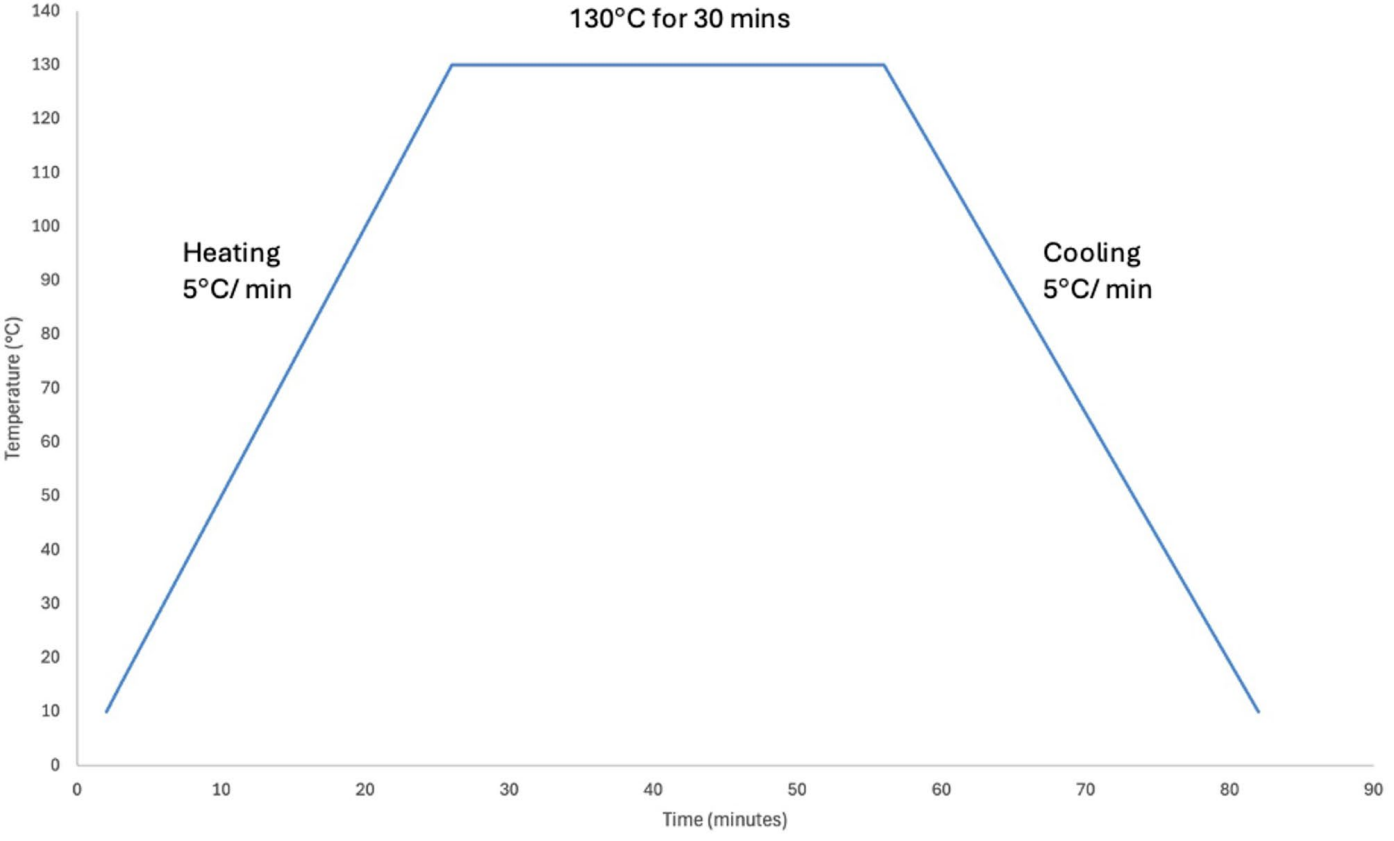
Dye procedure gradient diagram.

The fabrics were dyed using fluorescent disperse dye TeRasilâ Red 3BFF (Huntsman, UK) at a concentration of 2%, 1% and 0.5% (Table 3). TeRasilâ is a classic disperse dye suitable for dyeing polyester and is commonly used for high-visibility workwear/sportswear, fulfilling EN 471 and EN 1150 standards for high visibility and/ or protective workwear which is classified under Colour Index Disperse Red 362 (Huntsman, n.d). To maintain parameter consistency, all 12 fabric swatches were placed into individual beaker dyeing apparatus (Mathis Labomat, Mathis, Germany) under a manual programme reaching 130 °C for 30 min at 5 °C/min heating and cooling gradient and a maximum pressure of 8 bar (shown in Fig. 9). The control fabrics were kept undyed and placed within filtered water only, this was to isolate the variable of microfibre identification and the methodology from that of the dyeing process.

It is assumed that commercially used dyeing processes could influence microfibre release due to the use of high temperatures, pressures and use of other chemicals and additives^54^. However, as the techniques and dye used does not differ from commercially used or available products/ methods, this study does not assume any greater microfibre shedding than commercially available textiles using similar techniques. For this study, the purpose of including undyed controls was to evaluate the methods used for fibre illumination and quantification, rather than the effects introduced by the dyeing process itself.

Following the dyeing process, reduction clearing was carried out by treating the dyed polyester samples in an aqueous solution containing sodium hydrosulphite and sodium hydroxide at 70–80 °C for 15 min, followed by hot and cold-water rinsing totalling 3 min. The water used was filtered distilled water to remove potential contamination, and the chemical reduction time was kept constant for each sample despite varying concentrations of dyeing to keep parameters the same and not have excess washing interfere with microfibre release within the wash tests. This process chemically reduces and solubilizes unfixed disperse dye molecules on the fiber surface, allowing them to be removed from the fabric. Following this, the swatches were rinsed with distilled water using a pressurised wash bottle (Alzon, Scientific Laboratory Supplies, UK) to remove contamination from the production, dyeing process or post-treatment processes of the fabric swatches.

Once the dyed fabrics were dried within in a fume cupboard, the pre-wash weight of each fabric swatch was documented using a balance with an accuracy of 0.1 mg (PS-60, Fischer, UK). The fabric swatches were wrapped individually in foil to reduce airborne contamination during the movement or storage of the fabric swatches.

**Table 2:** Fabric specifications.

Structure	Yarn type	Fabric sample size (cm)	Mass (g)	Thickness (mm)
Single Jersey knit	Undyed intermingled polyester 2ply 167 Decitex with 48 filaments per end, 100 turns per meter spun counterclockwise	9 by 9	1.72	1.38

**Table 3:** Dye specifications and procedure.

Dye	Dye concentration	Dye measurements	Dyeing procedure	Replicates
TeRasilâ Red 3BFF	2%	1 g dye with 50mL filtered water	130 °C for 30 min and a 5 °C/min heating and cooling gradient and a maximum pressure of 8 bar	3
TeRasilâ Red 3BFF	1%	0.5 g dye with 50mL filtered water	130 °C for 30 min and a 5 °C/min heating and cooling gradient and a maximum pressure of 8 bar	3
TeRasilâ Red 3BFF	0.5%	0.25 g dye with 50mL filtered water	130 °C for 30 min and a 5 °C/min heating and cooling gradient and a maximum pressure of 8 bar	3
Undyed	Undyed	50 mL filtered water	130 °C for 30 min and a 5 °C/min heating and cooling gradient and a maximum pressure of 8 bar	3

### Washing and filtration procedure

The fabric swatches were washed in individual steel canisters placed in a laboratory wash simulator (Washtec, Roaches UK) using ISO4484-1:2023 standardised wash test procedure (BSI, 2023). Briefly, 50 stainless steel balls (⌀6 mm) and 360mL of distilled water were added alongside the test fabric swatches into individual stainless-steel containers for 45 min at 40 °C.

Following the wash simulation the test liquor was filtered onto pre-rinsed, dried and weighted Whatman GF/F membrane (1.2 μm pore size, 50 mm diameter) using a glass Bucher funnel and vacuum pump. The container, stainless steel balls and fabric were rinsed three times with distilled water within a pressurised wash bottle (Alzon, Scientific Laboratory Supplies, UK) to ensure that all loose fibres from the fabric and container were located onto the filter membrane. After filtration, a final rinse of the edges of the Buchner funnel was conducted with distilled water from the pressurised wash bottle to ensure no fibres had adhered to the edges of the funnel. Filter membranes were placed into individual glass petri dishes to dry and kept within a laminar flow cabinet.

### Microfibre characterisation

Quantification of fibres was initially acquired gravimetrically, with differences in the dry filter membranes assessed before and after filtering (Eq. 1, adapted from BSI^19^.

1 $$\:Mmf={Fm}_{2}-\:{Fm}_{1}$$

$\:\mathrm{Where\:}\text{}$ $\:Mmf$ is the mass of microfibres released expressed in mg;

$\:{Fm}_{2}$ is the mass of the filter after the wash test, expressed in mg;

$\:{Fm}_{1}$ is the mass of the filter before the wash test, expressed in mg.

Following this, the mass of microfibres released is calculated as a ratio of the mass of fabric, showing as microfibres released (mg) per kg of fabric (Eq. 2, adapted from BSI^19^.

2 $$\:Rf=\:\frac{Mmf}{Mfs}$$

Where.

$\:Rf$ is the ratio of mass of the microfibres released related to mass of fabric sample, expressed in mg per kg;

$\:Mmf\:$ is the mass of microfibres released expressed in mg;

$\:Mfs\:$ is the mass in kg of the fabric sample.

**Fig. 10:**
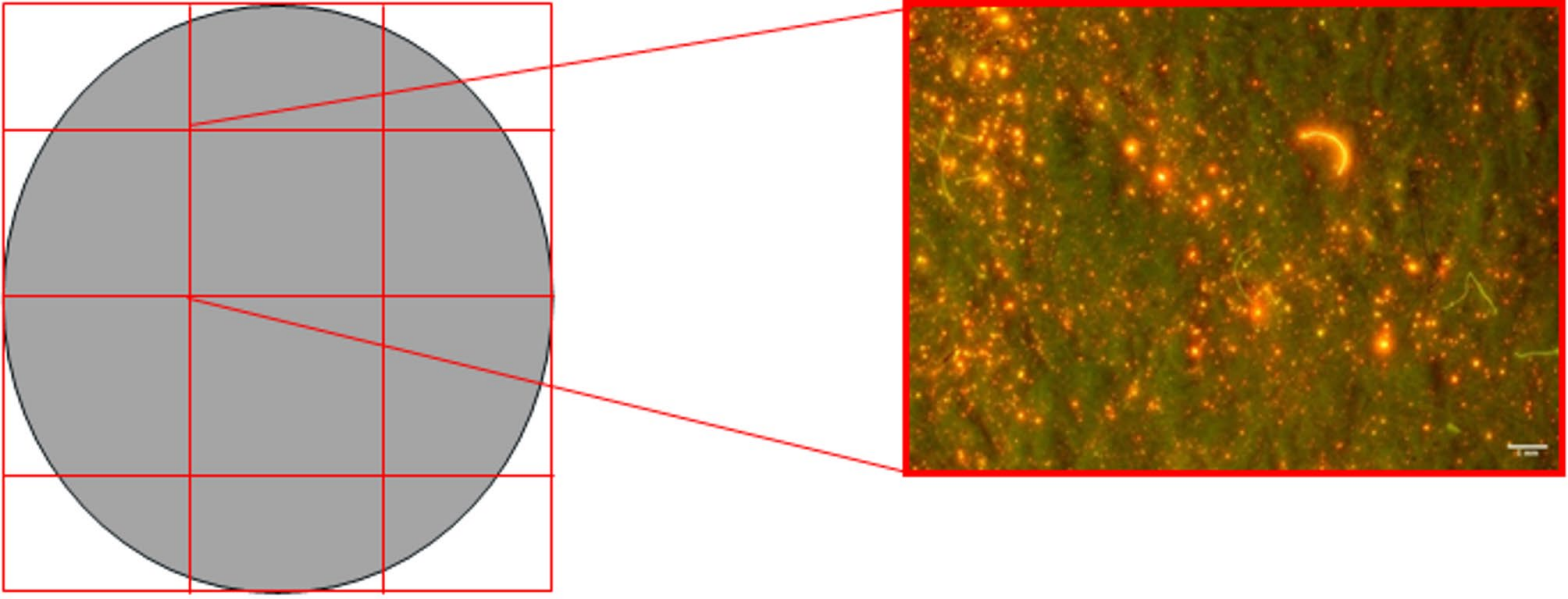
Filter membrane area analysed by optical microscopy under semi-automated analysis. (Authors’ own).

Images of the filter and shed microfibres were obtained using a digital microscope (Canon 4000D, Canon, UK) fitted with a macro lens (Canon MP-E 65 mm f/2.8 1-5x/ Canon, UK) in accordance with methods reported by Kukkola et al.^60^ and Maes et al.^16^. Briefly, the camera is operated on a CNC machine using Mach3mill software (Artsoft, Newfangled Solutions) to automatically take 12 images of the 50 mm filter membrane (Fig. 10, full specifications provided in the supplementary information Figure S1, Text S1). Whilst the movement of the camera to take the images is automated, care was taken to ensure areas of the images were in focus as this could impact the process of microfibre analysis. Each image has a resolution of 5184 × 3456 pixels with a resolution of 0.236 μm/pixel.

Several images were also acquired using a Nikon Digital Camera DXM1200F with TRITC filter cube (Ex: 540 nm, Nikon Instruments Inc, UK) to assess validity of the methodology.

Image contrasts and brightness were adjusted with aperture, shutter speed and ISO of the camera primarily, followed by fine tuning within ImageJ (open-source image processing software, version 2.9.0/1.53t). For experimental opportunities, fibre quantification and lengths of shed microfibres were obtained via ImageJ and through MATLAB software (MATLAB_R2023b with the Image Processing Toolbox add-on). This is explained further within the supplementary document with full MATLAB code and steps taken within ImageJ software.

### Statistical analysis

Significant differences between data acquired for the weights of the filter membranes of the different dye concentrations was analysed by an unpaired T-Test as performed between undyed and 0.5% dye concentration, between 0.5% and 1% and between 1% and 2%. A significant difference between weights was identified with *p* values < 0.5.

For the microfibres identified under ambient and fluorescent light, a two-way ANOVA test was performed to examine statistical differences between quantity of microfibres identified under ambient and fluorescent light, as well as differences between the sizes of identified microfibres. A statistically significant difference was identified with *p* values < 0.5%.

Contamination controls.

To reduce contamination, all equipment was triplicate rinsed with distilled water and stored within a laminar flow cabinet low in airborne microfibre contamination. The imaging took place in a custom-built blackout box, which blocked out light to optimise imaging, but also to reduce atmospheric contamination. Additionally, procedures from Woodall et al.^61^ were implemented such as cleaning of the surfaces, minimising air exposure to samples, wearing cotton lab coats and gloves to try and protect samples from researchers and clothing contamination. However, due to the history of the laboratory being heavily used for textile research, and within the same premises as the in-house knit laboratories, and mimicking that of textile testing facilities within the textile and apparel industry, residual contamination was expected and thus control samples were undertaken to measure contamination levels. The abundance and colour of the captured contamination was analysed. The control samples were used to progress the analysis techniques used for the test samples, ensuring that the semi-automated counting did not ‘pick up’ the contamination.

## Data Availability

Data will be made available on request, please contact corresponding author.
